# Engagement of non-government organisations and community care workers in collaborative TB/HIV activities including prevention of mother to child transmission in South Africa: Opportunities and challenges

**DOI:** 10.1186/1472-6963-12-233

**Published:** 2012-08-02

**Authors:** Jeannine Uwimana, Christina Zarowsky, Harry Hausler, Debra Jackson

**Affiliations:** 1 School of Public Health, University of the Western Cape, Modderdam Road, Bellville, 7535, Cape Town, South Africa; 2 TB/HIV Care Association, 25 St. George Mall, Cape Town, 7441, South Africa

**Keywords:** Participation, Community care workers, Collaborative TB/HIV activities, South Africa

## Abstract

**Background:**

The implementation of collaborative TB/HIV activities may help to mitigate the impact of the dual epidemic on patients and communities. Such implementation requires integrated interventions across facilities and levels of government, and with communities. Engaging Community Care Workers (CCWs) in the delivery of integrated TB/HIV services may enhance universal coverage and treatment outcomes, and address human resource needs in sub-Saharan Africa.

**Methods:**

Using pre-intervention research in Sisonke district, KwaZulu-Natal, South Africa as a case study, we report on three study objectives: (1) to determine the extent of the engagement of NGOs and CCWs in the implementation of collaborative TB/HIV including PMTCT; (2) to identify constraints related to provision of TB/HIV/PMTCT integrated care at community level; and (3) to explore ways of enhancing the engagement of CCWs to provide integrated TB/HIV/PMTCT services. Our mixed method study included facility and NGO audits, a household survey (n = 3867), 33 key informant interviews with provincial, district, facility, and NGO managers, and six CCW and patient focus group discussions.

**Results:**

Most contracted NGOs were providing TB or HIV support and care with little support for PMTCT. Only 11% of facilities’ TB and HIV patients needing care and support at the community level were receiving support from CCWs. Only 2% of pregnant women reported being counseled by CCWs on infant feeding options and HIV testing. Most facilities (83%) did not have any structural linkage with NGOs. Major constraints identified were system-related: structural, organizational and managerial constraints; inadequate CCW training and supervision; limited scope of CCW practice; inadequate funding; and inconsistency in supplies and equipment. Individual and community factors, such as lack of disclosure, stigma related to HIV, and cultural beliefs were also identified as constraints.

**Conclusions:**

NGO/CCW engagement in the implementation of collaborative TB/HIV/PMTCT activities is sub-optimal, despite its potential benefits. Effective interventions that address contextual and health systems challenges are required. These should combine systematic skills-building, an enhanced scope of practice and consistent CCW supervision with a reliable referral and monitoring and evaluation system.

## Background

The dual burden of TB and HIV in sub-Saharan Africa raises many questions on how best to address issues surrounding access, equity, and quality of healthcare, service delivery mechanisms, and the strengthening of health systems. The World Health Organization (WHO) and other international bodies have been advocating for the implementation of collaborative TB/HIV activities as a strategy to mitigate the impact of the dual epidemics on disease programmes, health systems, patients, and communities at large [1]. The 2004 WHO-Stop TB Partnership’s interim policy for collaborative TB/HIV activities stressed the importance of addressing the TB and HIV epidemics across disciplines and agency lines, and of engaging community members [1]. This echoes the wide recognition of community participation in health as a critical component of Primary Health Care (PHC) since the Alma Ata declaration in 1978; however, the realization of this vision has been limited [2,3].

The high demands for HIV- and TB-related care and support exceed the human resource capacities in many developing country health systems, including South Africa [2]. The literature reflects the increasing contribution of non-governmental organisations (NGOs) and Community Care Workers (CCWs) such as community health workers (CHWs) and home based carers (HBCs) in community health, through improving health service coverage, efficiency, effectiveness, equity, access and self-reliance, including among people with HIV and TB [4-6]. The term “Community care workers” is used in this study to denote all categories of “non-professional” health workers such as CHWs, HBCs, TB DOT supporters, and others, as CCW involvement in collaborative TB/HIV activities can include a range of interventions: screening for TB symptoms, home-based HIV counseling and testing (HCT), TB directly observed therapy (DOT) support, antiretroviral therapy (ART) adherence support, education on PMTCT (including feeding options), home-based care, advocacy, and community mobilisation [4,5,7,8]. Such interventions have the potential to prevent infection, identify new cases, manage illness, and improve the continuity of care, which together could lessen the impact of TB and HIV in the community at large [9].

In South Africa, interest in engaging CCWs (particularly CHWs and HBCs) has increased, largely due to the human resources challenges of addressing the dual TB/HIV burden [7,8]. Increased demand for additional and lower cost health workers has led to large numbers of NGOs becoming involved in both HIV and TB programmes. Many cadres of CCWs have been created, including: lay HCT counselors; generalist CHW focusing on health promotion; HBCs who provide palliative care to terminally ill AIDS clients; youth ambassadors (health education on HIV prevention among youth); and TB DOT supporters. This proliferation of cadres and positions has created jobs (albeit low-paying) in a context of very high country-wide unemployment. It has also helped mitigate the impact of the epidemic in South Africa [2,10]. However, these multiple CCW cadres are generally trained in a single disease or programme dictated by the NGO or funder [10]. The result is a “specialized CCW” model rather than the comprehensive or generalist approach intended by the national policy framework [11].

To further complicate the situation, the dominant CCW management model in South Africa at the time of this study was of government contracts with NGOs, who in turn employ and deploy CCWs. Since 1985, KZN province has had a strong CHW programme supported by local government, funded through national budgets [5]. With the explosion of HIV and TB, additional cadres and costs such as training were covered by external funders.

At the time of this study, the KZN Department of Health had contracted a single NGO to employ and manage all CHWs across the province, while 135 NGOs were contracted to employ and manage several other cadres of CCWs. Additionally, other NGOs not funded by the DOH also managed HBCs. The NGO managing CHWs was overseen at the provincial level, while the other NGOs were managed at the district level.

At the community level, this model has reinforced the provision of structurally separateTB, HIV, and PMTCT services. This separation has created conflicts among CCWs and discomfort to patients who are visited by several CCWs [7,10]. CCWs have great potential for expanding primary health care (PHC) by integrating these services, thereby bridging the current service delivery gaps in vertical TB, HIV and PMTCT programmes, yet in most settings they are under-utilized [12].

To date, few empirical studies have assessed the level of NGO and CCW engagement in collaborative TB, HIV and PMTCT activities or explored ways CCWs could provide integrated TB/HIV/PMTCT care at the community level. In this paper, we report on a sub-study of a larger project on community participation in enhancing collaborative TB/HIV/PMTCT programmes in a rural district of KwaZulu Natal (KZN), South Africa. We report on three study objectives: (1) to determine the extent of the engagement of NGOs and CCWs in the implementation of collaborative TB/HIV including PMTCT; (2) to identify constraints related to provision of TB/HIV/PMTCT integrated care at community level; and (3) to explore ways of enhancing the engagement of CCWs to provide integrated TB/HIV/PMTCT services.

## Methods

### Setting

KwaZulu-Natal is the epicenter of TB and HIV epidemics with a TB-HIV co-infection rate of 75-80% in some settings [13]. Sisonke district, one of KZN’s 11 districts, is mostly rural with poor roads, an area of 11,128 km², a population of ~500,000, 79% unemployment, and poverty levels of 71%. The people of Sisonke District have relatively poor access to basic services even compared to residents of similar rural-inland districts; only 33% of Sisonke residents have access to piped water, on or off site, 57% rely on candles for lighting, 74% are reliant on either paraffin or wood for cooking and only 22% have access to good sanitation, flush or chemical toilets. IsiZulu is the most spoken language and the majority of the district population (53.62%) are females compared to males (46.38%). The antenatal HIV prevalence is estimated at 35% compared to 39.5 province-wide 2008–2009 [14]. New TB cases numbered 1079 per 100,000 population with an HIV co-infection rate of 81%, compared to 52% overall in SA in 2009 [15].

At the time of the study (August-2008 to Sept-2009), the district had a total of 32 NGOs managing HBCs, 26 of which were funded by the DOH. These 26 NGOs employed a total of 414 HBCs. Another large independent NGO managed 402 CHWs. Therefore, collaborating with these 33 NGOs provided us access to a total of 816 CCWs (CHWs and HBCs).

### Ethical considerations

Ethical clearance was obtained from the University of the Western Cape and the DOH-KZN Research Unit Institutional Review Board. We also received permission from the Sisonke District Manager and health facilities managers to access patient records and other related data.

An information sheet and consent form addressing voluntary participation in the study, anonymity and confidentiality was reviewed with all participants. The household survey was not specific to HIV or TB, but all participants understood that questions on TB and HIV would be asked and that they were free to disclose or not disclose their household’s history of TB, HIV, and related services.

### Study design

A mixed methods approach was used with a partially mixed concurrent dominant status design [16] by exploring specific objectives through each method in order to achieve a more comprehensive view on issues related to community engagement in healthcare. Quantitative research methods were used to answer objective 1 while qualitative methods were used for objectives 2 &3. Quantitative and qualitative methods occurred concurrently. The quantitative facet was given more weight (dominant status) [16]. The quantitative and qualitative data were analysed separately and were integrated during interpretation.

### Study population and data collection

Participation in the study was voluntary and all participants provided written informed consent. Different recruitment strategies and data collection tools were used for different groups of participants in the study as illustrated in Table 1. Facilities providing PHC services, NGOs sub-contracted by the DOH-KZN to manage CCWs (CHWs and HBCs), CCWs, and household members from the facility and NGO catchment areas in Sisonke District were included in the study. Insert Table 1.

**Table 1:** Study objectives, Research methods and Sample size

**Objectives**	**Research method**	**Description**	**Sample size**
Objective 1	Quantitative	Facility audit :	
		Facility records review – registers and	42 District hospitals- 5 CHC and PHC facilities
To determine the extent of the engagement of NGOs and CCWs in the implementation of collaborative TB/HIV including PMTCT			
		*DHIS	
		Interview with facility managers using a self-administered questionnaire	37 facility managers
		NGO audit :	32 NGOs managing HBCs
		NGO records review and interviews with managers	1 NGO managing CHWs
			
		Household survey which included participant	n = 3,867 household members randomly selected from the catchment areas of audited facilities and NGOs
		aged 18 years and above	
Objective 2	Qualitative	Individual in-depth interviews with	29 Provincial managers – 6 District managers – 6 Facility managers – 11 NGO managers- 6 FGDs with CCW- 4 FGDs with patients- 2
		**key informants (Provincial and district managers; Hospital and facility managers; and NGO managers)	
Identify constraints related to provision of TB/HIV/PMTCT integrated care at community level			
		Focus group discussion (FGDs) with CCWs and TB,HIV and PMTCT patients	
Objective 3			
Explore ways of enhancing the engagement of CCWs to provide integrated TB/HIV/PMTCT services.			

*DHIS: District health information system.

**Key informants included provincial, district, and facility managers as well NGO managers.

#### Quantitative study

The quantitative aspect of the investigation comprised a facility and NGO audit and a household survey. The purpose of the audit was to determine the burden of disease managed by the facilities, assess linkages between facilities and NGOs in the provision of care and support related to TB/HIV/PMTCT, and document training levels of CCWs, The audit included records reviews and interviews. Facility records were reviewed to determine the burden of TB and HIV diseases and assess the continuum of care. The facility records review consisted of reviewing TB, pre-ART and ART and ANC/PMTCT registers of quarter 1 to quarter 4, 2008–2009 and the data from the district health information system (DHIS) from the same period. The extracted data was validated with respective facilities to ensure accuracy of the data. To complement facility records review, questionnaires for facility and NGO managers were designed by the researchers and presented to the district task team established to monitor the audit in order to assess the face validity (i.e., whether the questions make sense as a measure of a construct in the judgment of others) and practicality (i.e., likelihood to be successfully understood). This was done prior to data collection in the field. An independent NGO, TADSA, was contracted to conduct the audit in collaboration with the researchers. Self-administered questionnaires were provided to hospital and operational managers of all 42 health facilities and the 33 NGOs mentioned above. The Audit was conducted in Oct-Dec 2008.

A household survey was conducted in August 2009. Its purpose was to describe the socio-demographic characteristics, measure the burden of TB and HIV in the study communities, and examine the services reported to be provided by CCWs (CHWs and HBCs) in the community in order to inform the design and the evaluation of a community based intervention.

The survey was not exclusively studying TB and HIV, but this paper reports on a sub-study of a larger project, hence only the household data related to TB, HIV and PMTCT services provided by CCWs are discussed. A sample size of 2400 respondents was calculated using Epiinfo based on the total population of the catchment areas of 11 facilities selected from the 42 audited ones. The criteria for selecting these 11 facilities and hence their catchment populations are described under “qualitative study” below. The sample size of 2400 was calculated for a 1-sided test with 80% power and a 5% significance level to detect an increase of HIV testing uptake from 32% to 40% post intervention over a period of 6 months. Approximately 20% was added for non response, making a total of 3000 household members. The questionnaire was developed by the first author and was programmed into the cell phones in collaboration with the service provider (Clyral Research console). Prior to field work, 35 field workers were recruited and trained on the purpose of the study, the content and use of the cell-phones, and ethical issues.

After training of field workers, a pilot was conducted in 50 households in one village outside the study area and amendments were made to the instrument. The data generated from the household survey on the services related to TB and HIV including PMTCT was used to deepen understanding of the facility and NGO audits.

#### Qualitative study

The qualitative investigation comprised of 29 key informant interviews (KIIs) and 6 focus group discussions (FGDs). A purposive sample was constructed to capture a range of perspectives across different managerial levels, roles and responsibilities.

We conducted 29 KIIs with provincial, district and facility managers as well as NGO managers involved in TB and HIV care. Six focus groups discussions (FGDs) were also held with CCWs (4 FGDs) and TB and HIV clients (2 FGDs), as illustrated in Table 1.

Eleven facilities from among the 42 audited facilities were purposively selected to ensure that all levels of care were represented (district hospital, community health centre and PHC clinic) and to include facilities with high numbers of TB and HIV clients. 6 NGOs were also purposively selected from the 33 NGOs audited. CCWs and TB and HIV clients who participated in the FGDs were from the selected NGOs.

The key informant interviews and focus groups for both CCWs and beneficiaries focused on current service provision and strategies to engage NGOs and CCWs in collaborative TB/HIV activities, notably which core activities to provide within households. Our goal was to understand perceived constraints for engaging NGOs and CCWs in collaborative TB/HIV/PMTCT activities and perspectives of how CCWs could best be utilised and their services integrated.

The interview guides for KIIs and FGDs were developed in English by the first author (JU) and reviewed by DJ and HH. Individual interviews were conducted in English by JU since managers were conversant in English. FGDs with CCWs and clients were conducted in isiZulu by a research assistant. Interviews were recorded using a digital recorder consented by the participants and the transcripts were transcribed verbatim by an independent transcriber. Transcripts from FGDs were verbally translated and audiorecorded back into English and transcribed. The interviews and FGDs were conducted from October to December 2008.

### Data analysis

Quantitative data from facility and NGO audits were captured in Excel and analysed using SPSS version 19. The data from the household survey was captured using cell-phone exported in an Excel file and analysed using SPSS version 19. Measures of central tendency such as frequency, mean, proportion and standard deviation were used for both datasets (audits and household survey).

For qualitative study, a total of 33 transcriptions were captured, coded and thematically analysed, using a constant comparison process supported by ATLAS.ti software [17]. An analytical grid of key themes was developed based on the study aims and familiarization with the first few transcripts, and then applied to the rest of the transcripts, following the approach described by Pope and colleagues [18]. Triangulation and member checking were used to enhance rigour. Member checking occurred through meetings with the district management team and stakeholder workshops to feedback preliminary results and inform further analysis [19].

## Results

### Coverage, awareness, and TB/HIV/PMTCT services provided by NGOs

Facility records review showed that TB and HIV cases accounted for more than 50% (16,280 of 31,346 patient records) of the disease burden in the audited facilities in 2008. Only 11% of the 16,280 TB and HIV patients were recorded to be receiving support from NGOs/CCWs. Just 40% of facility managers reported being aware of NGOs operating in their catchment areas and could identify the village or ward where these CCWs are located.

The most commonly reported TB and HIV/AIDS services provided by the 33 audited NGOs included general HIV counseling (45%), TB symptom screening (39%), ART adherence support (36%) and condom distribution (33%). None of the NGOs provided adherence support to HIV-positive pregnant women on dual therapy. Table 2 illustrates the reported services provided by NGOs.

**Table 2:** Services related to TB/HIV care provided by NGOs and CCWs

**Service provided by NGOs/CCWs related to TB and HIV care**	**Number of NGOs(n)**	**Percentage (%)**
	N = 33	
General HIV education and HIV counseling	15	45.5
TB symptoms screening	13	39.3
Nutrition	13	39.3
ART adherence support	12	36.4
Support groups for HIV/AIDS patients	10	30.3
Condom distribution	11	33.3
ART defaulter tracing	6	18.1
Collection of TB medication	5	15.1
HIV counseling to pregnant women	5	15.1
TB defaulter tracing	4	12.1
TB DOT	2	6
Sputum collection	2	6
TB contact tracing	1	3.3
Streptomycin injections	1	3.3
Treatment adherence support to pregnant women	0	0

Of 3,867 household members interviewed, 78% (n = 3,012) were visited by a CCW (either CHW or HBC, or both) in the previous year. Nearly all (95%) who received a visit reported being counselled on health-related issues, 21% were screened for TB symptoms, 20% received HIV counselling, 7% were given TB treatment support, and 2% received ART adherence support. Few pregnant women (2%) reported being counselled on infant feeding and HIV testing. Table 3 summarises the proportion of households served by CCWs and the types of services reported to have been provided.

**Table 3:** Proportion of households served by CCWs and type of services provided

**Services provided**	**Number (n)**	**Percentage**
		(%)
Health education	2860	95
Screening of TB symptoms	645	21
Awareness on HIV counselling and testing	593	20
Home based HIV counselling and testing	1105	17
Collecting a sputum	338	11
Adherence support for TB treatment	214	7
Nutrition/food parcel	67	2.2
Adherence support for ART	61	2
Counselling on infant feeding	46	1.5
Adherence PMTCT clients	17	0.6
n = 3012		

### Linkages between facilities and NGOs

Seventeen percent of facilities had both an NGO referral mechanism and a standardised referral tool in place, while 42% of facilities reported not having anyone assigned to coordinate CCWs and ensure a linkage between health facilities and NGOs.

### Training provided to community care workers (CCWs)

The audit revealed that the majority of NGOs (67%) reported providing training of their CCWs on home based care, while only 21% offered training on HIV prevention and care, 15% on HCT, 12% on TB symptoms screening and TB DOT, and 2% on ART adherence and STIs symptoms screening. None of the NGOs reported offering any training on PMTCT, although 16% of NGOs offered training related to first aid, child and youth care, community development and leadership.

### Constraints for engagement of NGOs and CCWs in collaborative TB/HIV/PMTCT activities

The predominant themes that emerged related to perceived barriers for engagement of NGOs and CCWs, including structural and managerial factors, training and skills needed by CCWs, lack of financing for community-based activities, and individual and community factors.

### Structural and managerial factors

The two main issues that emerged from participants in this category included (1) a lack of structured linkages between health facilities and NGOs, and (2) inadequate supervision of CCWs.

#### Lack of structured linkages between facilities and NGOs

In South Africa, the government is the primary funder of CCWs through NGOs, even though CCWs are not part of the formal health system and are managed by subcontracted NGOs which also often receive complementary funding directly from donors. Although most of PHC clinics are complemented by CCWs, there is no formal structural mechanism that links NGOs-CCWs with PHC clinics. This lack of structured linkages between facilities and NGOs-CCWs was perceived as a major constraint. Managers from both provincial and district levels strongly believed that facilities should have set mechanisms for linking to community-based activities. Further, the responsibility for such structures and mechanisms was believed to belong to the district and facilities.

"”*There is no proper link between health facilities and CCWs…there is no structure in place and no one in the facility cares about having that sort of mechanism for coordination. It is the mandate of districts and facility managers and NGOs.” (PM)*^a^"

However, district managers suggested provincial managers did not provide the support to establish such structures; for example, even when facilities were willing, the district did not provide the necessary funding.

"*“There is a need of a referral system in place that can improve a lot …but well you cannot plan in the air … we don’t have any funding so we really don’t know what is happening with these people [CHWs, HBCs].” (FM)*"

Provincial and district managers also argued that it is the responsibility of facility managers to establish structures. However, facility managers disagreed, noting that CHWs have always been managed at district and province levels. When NGOs began HIV and TB programmes, no link existed between the NGOs and individual health facilities; NGOs were co-managed centrally by the district and province. Thus, it is not surprising that the facility audit found that more than half (60%) of facilities could not say how many NGOs are in their catchment area or what they do. Communication and collaboration between these groups are clearly insufficient.

#### Inadequate supervision of CCWs

Both government and NGO managers acknowledged that a lack of CCW supervision has been an obstacle for quality service provision in the community. Although NGOs had staff assigned to supervise their CHWs and HBCs, the large numbers of CCWs have made supervision problematic. Participants expressed the need for establishing a supervision structure at the health facility level to enhance the supervision of CCWs.

"*“You could see that things are going wrong but… It’s just lack of proper supervision; we need a person, a post at facility level, institution level who will be in charge of community health workers.” (FM)*"

### Training of CCWs and deficiency in skills

Most CCWs are trained according to the mandates of their employing NGO, and for narrowly defined specific tasks. CCWs (CHWs and HBCs) felt that they should all be trained to provide multiple services, as noted in one of the focus groups.

"*“Our training should be the same and all carry the same name in the community. The community cannot differentiate between the CHWs and HBCs. HBCs should just become the CHWs, this would help us”. (FGD, 1)*"

Few NGOs offered their CCWs any training related to PMTCT, and most offered limited and very task-specific training related to TB and HIV. This lack of training has been influenced by donor funds and to a lesser extent, by programme managers who currently run TB and HIV trainings separately for different cadres of CCWs.

"*“Most CCWs are trained according to what the funders who are supporting a particular NGO want; hence these services are disease-based and verticalised, although we as a Department of Health would like to see these CCWs trained comprehensively.” (PM)*"

### Financing of community-based activities

Low CCW stipends and a lack of resources and equipment were identified by participants as barriers related to financing of community-based activities.

#### Remuneration and motivation of CCWs

Across all categories of participants, low and non-standard CHW and HBC stipends were identified as a factor that influenced CCW motivation, an issue also documented in the literature [7]. CHWs receive a stipend of Rand 1,500 (219 USD) per month while HBCs receive Rand 500 (73 USD).

"*“We have a special request when it comes to money because food is expensive and we are doing a lot of hard work. At the end of the month you find that you are going to get little money, that you are only going to buy food and then it’s gone. We don’t know how we will pay school fees for our children because they [Department of Health] also say we must not mix this job with any other. What are we going to do with a R1,500 while things are expensive? … it is not good… de-motivating.” (FGD, 1)*"

#### Lack of resources and equipment

Lack of transport for CCW supervisors and sparsely populated districts further compounds the problems with supervision. Also, frequent gaps in the supply of resources and equipment, such as home based care kits, were also mentioned as a hindrance for community service provision.

"*“I am a HBC, we usually have stumbling blocks…we don’t receive the home based kits and porridge for our clients. Sometimes we used to give bandages for the child if he /she is wounded but the bandages are no longer there.” (FGD, 1)*"

### Individual and community factors

While the system “supply side” factors were the predominant obstacles, sub-themes emerged across all categories of participants that included health care worker attitudes towards CCWs, HIV stigma in the community and lack of disclosure, and cultural beliefs and norms.

#### Health care worker attitudes towards CCWs

Most programme managers at the province and district levels, NGO managers, and CCWs felt that CCWs are not seen as part of the health system.

"*“Most of the professionals do not give credit to CHWs for the work they do and feel that they are not part of the health system” (FM)*"

While the study did not further explore the impact of such attitudes, research elsewhere stresses the detrimental effects of negative health worker attitudes on CCW credibility and on facility-community referrals [7]. It is likely that lack of recognition of CCWs as state employees and part of the formal health system have contributed to tension and poor relationships between CCWs and health care workers [2], and NGOs managing CCWs with facility management to a certain extent.

#### HIV stigma in the community and lack of disclosure

Most NGO managers and CCWs perceived HIV stigma as a challenge for HCT and HIV diagnosis disclosure. As one CCW put it,

"*“The main problem in the community is stigma. When you visit a household, [household members] will hide the person who is sick if they know that he/she is HIV positive or the client will not disclose his/her status....” (FGD, 1)*"

Some TB and HIV clients interviewed felt that they should disclose their status to CCWs so that they could receive needed support. However, there is some fear among HIV infected clients regarding lack of confidentiality and emotional preparedness.

"*“My opinion is that we must tell the CHWs everything… people are scared to tell local people because they always think that she is going to gossip about me and tell other people about my status. I think that people have that thought/mentality while illness is eating him instead of disclosing to the CHW and getting quick help.” (FGD, 2)*"

CCWs also expressed that cultural beliefs and norms can stop people from disclosing their HIV status and seeking care.

"*“People with HIV don’t disclose their HIV status, they hide themselves. Sometimes when you provide HIV education in a household and as a result, the clients disclose their HIV status, then afterward, they just say I was bewitched I have to go to an Inyanga [Traditional doctor].” (FGD, 1)*"

### How to engage NGOs and CCWs in collaborative TB/HIV/PMTCT activities at the community level

Actions to address the reported constraints emerged as the predominant themes in the interview data,: the integration of diverse community care cadres into one cadre; harmonisation of scope of practice across cadres; training for all CCWs on comprehensive TB/HIV/PMTCT care provision; improved supervision for CCWs; and the implementation of a monitoring and evaluation (M&E) system for community-based NGO/CCW programmes.

#### Integration of diverse community cadres into one cadre and harmonise scope of practice of CCWs

Most of the participants perceived that the integration of different CCW cadres would reduce conflicts and multiple visits to a single household, saving both time and money.

"*“…so this one client must establish a rapport with five different categories of CCW coming to the house whereas if it was just one person it would work much better. Even for us as government it’s not cost-effective because we have to pay three different people instead of one.” (DM)*"

"*“It is better if we [CHWs and HBCs] work together because people in the community do not like it if there are many people entering one household.” (FGD, 1)*"

Beyond the integration of various CCW cadres, some managers felt that CCWs should be comprehensive or generalists rather than specialists. They recommended merging the CCWs’ responsibilities and expanding their scope of practice.

"“*if you look at the history of CHW programme, the programme had CHWs who look at prevention and health promotion and HBCs who focus on palliative care. We are trying to move away from the old CHW programme where these CCWs should be one and harmonise their scope of practice. So it must be comprehensive.” (DM)*"

#### Training of CCWs to provide comprehensive TB/HIV/PMTCT care

All participants felt that CCWs should be trained comprehensively in order to provide TB/HIV/PMTCT integrated care. Managers identified many activities related to TB/HIV/PMTCT on which CCWs should be trained, including health education, TB case finding, home based HCT, treatment adherence, counseling on feeding options, and education on living with HIV. Table 4 illustrates recommended core TB/HIV/PMTCT care activities to be provided at the community level.

**Table 4:** Core activities for provision of TB/HIV/PMTCT integrated care by CCWs at community level


Health Education related to TB,HIV and maternal and child care	
Home based care	
TB Case finding and treatment adherence	
· Screening of TB symptoms and collection of sputa	
“C*ommunity Care workers should be given resources such as sputum bottles so that they can use when identify a TB suspect”.(NGOM)*	
· TB treatment collection and DOT	
Defaulter tracing and TB contact tracing	
Home based HIV counseling and Testing (HBHCT)	
· HIV counseling and Testing	
*“ Some people don’t want go to the facility when referred by CCWs for HIV testing they want it to be provided at home”(DM)*	
· Ongoing counseling to enhance disclosure	
*“You need enough support and counseling before you go an HIV test because If you are able to accept it there is no problem to disclose when you get home. (FGD,2)*	
Screening for STIs symptoms	
Adherence support for ART and dual therapy	
Nutritional support	
*“There is no food to give to the sick people. The porridge also gives strength to HIV positive people” (FGD,1)*	
Referrals for social services/grant	

#### A monitoring and evaluation system for CCW programmes

Both government and NGO mangers perceived strengthening monitoring and evaluation (M&E) systems for community-based programmes, including CCW supervision, as a strategy to enhance both facility-community interactions and the collaboration of NGOs and CHWs in TB/HIV/PMTCT activities.

"*“There should be someone at the facility such as a community health facilitator to ensure the link between the facilities and the CCWs and standardise the monitoring and evaluation system for the CHW programme.” (FM)*"

## Discussion

Although there is some variability in the facility-NGO audit data compared to the household survey in terms of services provided by NGO through CCWs, both the audits and the household survey reported limited involvement of NGOs and CCWs in provision of integrated TB/HIV/PMTCT services, particularly for TB case finding, HCT, treatment adherence (ART and TB) and PMTCT, despite the large number of TB and HIV patients needing community level care. The fact that 78% of community respondents reported at least one CCW visit in the previous year suggests that there is at least broad – if not deep – coverage at the community level.

The limited care and support provided to patients needing assistance at the community level – only 11% of facilities’ TB and HIV patients are reported to have received care and support -- indicates inadequate linkages between facility and NGOs. This division has contributed to poor follow-up of patients at the household level once enrolled into care.

Limited provision of community level TB/HIV/PMTCT integrated services can be partly explained by contextual issues such as denial of the HIV epidemic [by the previous Mbeki government] and health systems barriers that are not particular to KZN but are found throughout the country and the continent at large. Lack of leadership, historical structural and managerial factors inherited from vertical TB and HIV programmes, and lack of community participation in collaborative TB and HIV activities are determinants of poor implementation in many countries of sub-Saharan Africa, including South Africa [8,10,11,20,21].

This study’s respondents, however, emphasized the on-the-ground organisational and managerial factors, notably non-existent or unfunded mechanisms to foster health facility-CCW-NGO-community linkages, training practices, remuneration and other financing choices, and community and individual factors as major constraints for the engagement of NGOs and CCWs in collaborative TB/HIV/PMTCT activities.

TB and HIV services continue to be provided separately in the community, due in part to lack of comprehensive and integrated training by TB, HIV and PMTCT programme coordinators who still operate independently, and also in part to targeted funding imperatives similar to those reported elsewhere [7,22-24]. Comprehensive and integrated training should be geared towards ensuring competence in core activities related to TB/HIV/PMTCT integrated care to be provided by CCWs. To provide comprehensive and integrated care that incorporates the core TB/HIV/PMTCT activities, the scope of practice for CCWs needs to be expanded to include activities such as home based HCT [25-27].

Studies in the literature have shown that weak supervision of CCWs and lack of an M&E system have contributed to poor quality of care provided by CCWs [5;10–11]. The findings of this study suggest that an enhanced M&E system for community-based activities with proper supervision for CCWs may offer an opportunity for improving the engagement of NGOs and CCWs in collaborative TB/HIV/PMTCT activities. Supportive facility-level structures to link health facilities and CCWs appear to be key. This could include employing a community health facilitator or team leader to provide supervisory support to CCWs. Strategies to improve the facility and community interface should be geared toward health care professional and CCW working relations.

Finally, this study found strong support for integration of diverse community care cadres into one cadre with an expanded and generalized scope of practice. This change will require strong leadership at district and facility levels to enhance the integration of CCWs in the PHC system, as envisaged in the country’s current priority of re-engineering of PHC. However, more research is required to understand how CCWs can best be integrated within the district health systems operationally, rather than theoretically.

This study is not free of limitations that should be noted. As the study was conducted only in one district out of 11 districts in KZN province, the statistical generalizability of the results are limited.

However, the inclusion of a wide range of participants from government, civil society (NGOs), CCW and consumers of healthcare (patients), the large sample size of the household survey, and the mixed methods design enhance the robustness of the findings and increase the potential generalisability of these findings across the province and similar rural settings.

## Conclusions

CCWs can help bridge significant gaps in integrated TB/HIV/PMTCT service delivery at the community level. This study identifies contextual and health systems challenges that effective CCW engagement in KZN as elsewhere will likely face. Effective interventions will address structural, organizational and managerial constraints as well as inadequate funding for community-based activities and CCW incentives. These interventions should combine systematic skills-building and consistent CCW supervision with a reliable referral and M&E system.

In South Africa the CCW programme is not only health-oriented but also a politically charged issue, with expectations that threaten the already overwhelmed CCWs’ activities [10]. Reform will require a review of the CCW programme including the scope of CCW practice and re-direction of resources based on what is expected from them. Although the findings of this study focus on collaborative TB/HIV/PMTCT activities, these findings may be relevant to other PHC programmes and other settings beyond South Africa.

## Endnotes

^a^Coding convention; Provincial managers (PM); District manager(DM); Facility managers (FM); NGO manager (NGOM) FGD with CCW (FGD,1); FGD with patients (FGD,2)

## Competing interests

The authors declare that they have no competing interests.

## Authors’ contributions

JU conceptualised the study protocol and manuscript, the implementation of the study as well data analysis and drafted the manuscript. DJ and HH participated in the conceptualisation of the study protocol and reviewed the various drafts of the manuscripts. CZ participated in the review and revision of various drafts of the text manuscript. All authors have seen and approved the final version.

## Pre-publication history

The pre-publication history for this paper can be accessed here:

http://www.biomedcentral.com/1472-6963/12/233/prepub
